# Does Thrombocyte Size Give Us an Idea about Thrombocytosis Etiology?

**DOI:** 10.1100/2012/598653

**Published:** 2012-09-10

**Authors:** Selami Kocak Toprak, Betul Erismis, Sema Karakus, Nazmiye Kursun, Aysegul Haberal, Mustafa Gurhan Ulusoy

**Affiliations:** ^1^Department of Hematology, Faculty of Medicine, Baskent University, 06490 Ankara, Turkey; ^2^Department of Biostatistics, Faculty of Medicine, Ankara University, 06100 Ankara, Turkey; ^3^Hematology Laboratory, Faculty of Medicine, Baskent University, 06490 Ankara, Turkey; ^4^Department of Plastic, Reconstructive and Aesthetic Surgery, Ankara Training and Research Hospital, 06080 Ankara, Turkey

## Abstract

In the presence of a pathogenetic mutation in JAK2 or MPL, a differential diagnosis of essential thrombocythemia (ET) from reactive causes is relatively simple. However, in patients with suspected ET who lack JAK2 and MPL mutations, the exclusion of secondary causes is especially important. The study was aimed to explore the clinical application of particularly mean platelet volume (MPV), hemoglobin, red blood cell indices, white blood cell, serum iron profile, and C-reactive protein level in the differential diagnosis of thrombocytosis. Medical records of 49 patients, consisting of reactive thrombocytosis (RT) and ET were retrospectively reviewed. The mean MPV level in RT group was 7.49 fL, and in ET group was 8.80 fL (*P* < 0.01). A cutoff point of <8.33 fL was found to have significant predictive value according to ROC curve analysis. This cutoff was associated with 83% positive predictive value (PPV) and 74% negative predictive value (NPV) in the diagnosis of ET and had a sensitivity of 65% and specificity of 89% for ET. Investigation of MPV is cheap, quick, and noninvasive, and may serve as a predictor of primary thrombocytosis. High sensitivity, specificity, PPV, and NPV enable this test an important tool and a possible surrogate marker in clinical practice.

## 1. Introduction

The average platelet (PLT) count in most clinical laboratories ranges from 150 × 10^9^/L to 350 × 10^9^/L or 450 × 10^9^/L, although the level for any individual is maintained within small limits from day to day [1]. The three most important physiopathologic reasons of thrombocytosis are clonal, including essential thrombocythemia (ET) and other chronic myeloproliferative disorders, familial, including hereditary cases of nonclonal myeloproliferation resulting from thrombopoietin and thrombopoietin receptor mutations, and reactive, where thrombocytosis occurs secondary to various acute and chronic clinical conditions [2]. ET has traditionally been a diagnosis of exclusion, requiring the absence of reactive conditions and other clonal disorders that may present with thrombocytosis [3]. In the presence of a pathogenetic mutation in *JAK2 V617F* or *MPL*, a differential diagnosis of ET from reactive causes is relatively simple. However, in patients with suspected ET who lack *JAK2 V617F* and *MPL* mutations, the exclusion of secondary causes is especially important.

 The study was designed to evaluate the clinical application of mean platelet volume (MPV), hemoglobin (Hb), red blood cell indices, white blood cell (WBC), serum iron profile, and C-reactive protein (CRP) level in the differential diagnosis of thrombocytosis.

## 2. Materials and Methods 

### 2.1. Patients

The medical records of 49 patients, diagnosed to have reactive thrombocytosis (RT) (group 1, *n* = 26) and ET (group 2, *n* = 23) between 2008 and 2009, were retrospectively reviewed (Table 1). The diagnosis of ET was made according to the 2008 World Health Organization (WHO) diagnostic criteria [4]. Reactive thrombocytosis was defined as a PLT count > 450 × 10^9^/L; we have included only cases of iron deficiency anemia (IDA). The diagnosis of IDA was based on Hb concentration less than 13.5 g/dL in male, less than 12 g/dL in female, mean corpuscular volume (MCV) less than 80 fL, ferritin concentration less than 15 mcg/L in male and less than 5 mcg/L in female [5]. Patients with acute blood loss and those requiring parenteral iron replacement therapy, chronic renal failure, hypertension, coronary vascular disease, diabetes mellitus, cigarette addiction, hyperlipidemia, coagulopathy, infectious disease, connective tissue disorders, anemia of chronic disease, and cancer were excluded as well as patients not responding to oral iron therapy during the ongoing process. Hemogram parameters, iron profile results, and CRP levels of two groups were analyzed.

 All test were performed in the morning, after 8 hours of fasting. All subjects were seated for 5 minutes before collection. Tourniquets were used and all collections were completed in less than 1 minute. Venous blood was collected into 2 mL ethylenediaminetetraacetic acid tube for red blood cell analysis and a 4 mL serum separation tube for biochemical analysis. Complete blood counts were determined using the Coulter LH 750 (Beckman Coulter, Miami, FL, USA) automated hematology analyzer. Iron levels were studied with the Ferrozine-iron complex spectrophotometry on a Hitachi 747-200 Chemistry Analyzer (Roche Diagnostics, Indianapolis, IN, USA). The serum ferritin was assessed using a sandwich immunoassay on an Access 2 immunoanalyser within a Dx automated system from Beckman Coulter (Brea, CA, USA). CRP levels were determined with a Dade Behring BN II nephelometer (Siemens, Marburg GmbH, Germany) using commercial kit (CardioPhase, Siemens, Marburg GmbH, Germany).

This retrospective study's protocol was approved by the Local Research Ethics Committee.

### 2.2. Statistical Analysis

Statistical analyses were performed with SPSS software for Windows (Statistical Product and Service Solutions, version 15.0, SSPS Inc, Chicago, IL, USA). Quantitative variables were expressed as mean values ± standard deviation (SD) for normally distributed data. These data were compared using the Mann-Whitney *u*-and-*t* test. Differences between the qualitative variables were evaluated using the chi-square test. The correlation of PLT counts and MPV with other variables, including Hb, MCV, WBC counts, CRP levels, serum iron, unsaturated iron-binding capacity, and ferritin, were analyzed by Pearson's correlation test. The sensitivity and specificity of MPV level for the diagnosis were calculated under various cutoff ranges, and the receiver operating characteristic (ROC) curves were drawn. All *P* values were based on a 2-tailed test of significance (*P* = .05).

## 3. Results

The mean PLT count in group 1 was 548.72 × 10^9^/L (range: 462–722 × 10^9^/L), and in group 2 was 973.82 × 10^9^/L (range: 524–2270 × 10^9^/L) (*P* < 0.001). The mean MPV level in group 1 was 7.49 fL (range: 5.59–11.39), and in group 2 was 8.80 fL (range: 6.15–11.60) (*P* < 0.01). The results are summarized in Tables 2 and 3.

 The normal MPV range was between 7 and 12 fL (Coulter LH 750, Beckman Coulter, Miami, FL, USA). There were 10 patients with MPV levels below 7 fL, and 16 patients with MPV levels above 7 fL for group 1. There were only 2 patients whose MPV levels were less than 7 fL, and 21 patients whose levels were over 7 fL in group 2 (*P* < 0.05).

**Figure 1:**
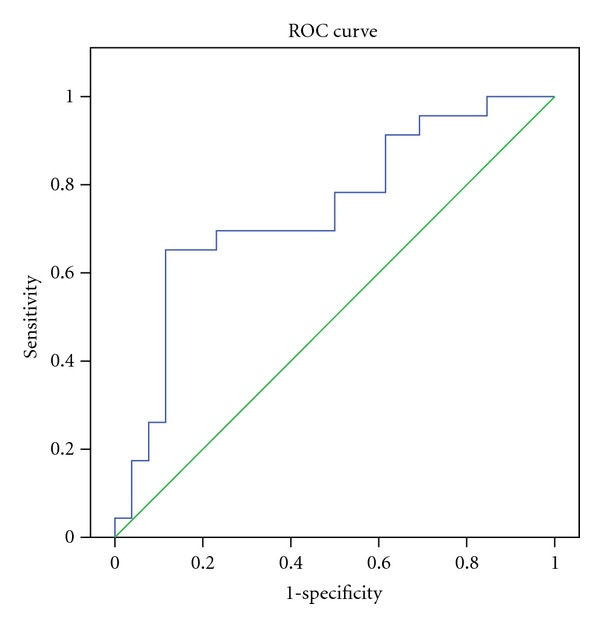
ROC curve analysis (*P* = 0,004 and area under the curve (AUC) = 0,742).

 A cutoff point of <8.33 fL was found to have significant predictive value according to ROC curve analysis. This cutoff was associated with 83% positive predictive value (PPV) and 74% negative predictive value (NPV) in the diagnosis of ET (odds ratio: 14.37; 95% CI: 3.28–63.00) and had a sensitivity of 65% and specificity of 89% for ET (Figure 1).

*JAK2 V617F* mutation was found in 13/23 patients in ET group (Table 1). The heterozygote patients population consisted of 7 women and 6 males aged between 19 and 87 years (mean: 58.08); nonmutated patients group consisted of 7 women and 3 males aged between 58 and 86 years (mean: 72.90) (*P* < 0.05). There was no statistically significant difference between two groups in terms of gender (*P* > 0.05). The mean PLT count in nonmutational patients group was 1030.00 × 10^9^/L (range: 626–2270 × 10^9^/L). The mean PLT count in heterozygote patients group was 961.96 × 10^9^/L (range: 524–1956 × 10^9^/L) (*P* > 0.05). The results are summarized in Table 4.

 When Pearson's correlation test was performed, inverse correlations between PLT counts, Hb, and MCV and on the other hand, linear correlation between PLT counts and WBC counts were observed in ET group. On the contrary, there was not any significant correlation between PLT counts and the other parameters including Hb, red blood cell indices, serum iron profile, and CRP in the reactive thrombocytosis group. Moreover, we did not found correlation between MPV and all the other parameters (Hb, red blood cell indices, serum iron profile, CRP, and WBC) in ET group; but, there were inverse correlations between MPV and serum iron, ferritin, Hb, and MCV in reactive thrombocytosis group. These results are presented in Tables 5 and 6.

## 4. Discussion

Differential diagnosis of thrombocytosis is not always obvious, since multiple causes may be involved. The routine clinical chemistry laboratory classically provides only limited help in distinguishing between reactive thrombocytosis and autonomous thrombocytosis, where PLT production escapes normal regulatory processes [6]. The peripheral blood smear test also gives information about the number and shape of PLTs. Moreover, the available means to differentiate these two entities are not specific enough. Some authors tried to distinguish thrombocytosis in ET from reactive thrombocytosis by using PLT parameters provided by blood analyzers [7, 8].

 The present study was designed in an attempt to characterize the different thrombocytosis states by PLT size, other hemogram parameters, serum iron profile, and CRP levels.

 MPV is a machine-calculated measurement of the average size of platelets. The MPV is generally increased in the myeloproliferative disease [9]. However, there is a nonlinear inverse relationship between the MPV and the PLT count within normal individual [10].

 We observed that MPV was significantly higher in ET than in RT. Similar results were reported by Osselaer et al. [6]. In the study, where 250 cases (RT: 174, myeloproliferative disease: 42, other: 34) with PLT counts of >500 × 10^9^/L were included, it was reported that MPV was significantly higher in patient groups compared to RT and also in both RT and ET groups MPV and PLT count show correlation [6]. However, in our study, no correlation between these two variables was found either in RT group or in ET group. On the contrary, in the study by Sehayek et al., while an inverse correlation was detected between MPV and PLT count, interestingly, MPV values in both the healthy control group and the RT group were found to be significantly higher compared to the ET group [7]. It has been demonstrated that small PLTs originate in megakaryocytes of a lesser ploidy than the larger ones [11]. In this regard, it is likely that in some cases of ET, a more prominent shift to the left in megakaryopoiesis is present, with the rate of proliferation of the megakaryocytes exceeding that of their maturation, resulting in an excess of megakaryocytes of lesser ploidy [7]. This would explain the considerable excess of small PLTs observed in ET. However, the percentage of microplatelet was also increased. It is probably a reflection of the megakaryocytic abnormalities that are often found in patients with myeloproliferative disease. Patients with RT also had an increased percentage of microplatelets but a lower number of megathrombocytes [12]. While in ET group, no variable was found to be correlated with MPV, in RT group an inverse correlation with Hb, MCV, serum iron, and ferritin levels was observed. Furthermore, the fact that in our study, the MPV was found lower in RT group is in agreement with the rule of higher PLT count and lower MPV association in normal individuals [10, 13].

 In the ET group (*n* = 23), the *JAK2 V617F* heterozygote mutant cases (*n* = 13) were similar in terms of sex, WBC, Hb, and MPV, to non mutated cases (*n* = 10) while the average age of the mutated group, was found to be significantly lower (Table 4). In our study, mean PLT count was found to be lower in mutated cases, although it did not reach statistical significance. On the contrary, the study by Hu et al. that included 145 myeloproliferative disease cases, showed higher WBC count and lower mean PLT count in *JAK2 V617F* positive patients, while age at diagnosis was higher [14]. In another study including 50 myeloproliferative disease cases, of which 26 were *JAK2 V617F* negative, it was detected that the age and Hb level were higher in patient bearing the mutation, but on the other hand, PLT count was significantly lower [15]. Literature data pointed out *JAK2 V617F* mutation is more common in older patients with myeloproliferative disease, as older patients have an higher allele burden [16].

 While in RT group, no correlation was found between the mean PLT count and any hematological variables, the positive correlation with WBC in clonal thrombocytosis group can be explained by the existence of a malign clonal proliferation. In fact, as expected, mean WBC count in ET group was found to be significantly higher, compared to RT group. In this group, PLT count also shows an inverse correlation with Hb and MCV. In a study by Abdulkarim et al. from Sweden, it was found that the only independent parameter affecting survival was lower Hb, despite WBC count was found higher than PLT count in ET group, compared to the healthy population [17]. In the same study, it was seen that higher transformation risk into acute myeloid leukemia was associated with a higher WBC count and a lower Hb level. In polycythemia vera (PV) on the other hand, a higher WBC count seems associated with an increased thrombotic risk as reported by Carobbio et al. [18].

## Figures and Tables

**Table 1:** The demographic characteristics of patients.

Diagnosis	Reactive thrombocytosis	Essential thrombocythemia	*P*
*n*	26	23	
Age	45.65 ± 15.77	65.04 ± 15.36	0,000
Sex (F/M)	16/10	14/9	>0.05

Reactive thrombocytosis: Etiology of iron deficiency anemia	Menorrhagia (*n* = 16, female)		
	Peptic ulcus (*n* = 1, male)		
	Gastric resection (*n* = 1, male)		
	Hemorrhoid (*n* = 8, male)		

Essential thrombocythemia: *JAK2 V617F* mutational status	Negative (*n* = 10)		Heterozygote mutant (*n* = 13)

**Table 2:** Comparison of hemoglobin, red blood cell indices, and serum iron profile.

	Group 1 (reactive thrombocytosis—IDA)	Group 2 (essential thrombocythemia)	*P*
Hemoglobin (g/dL)	8.89 ± 1.65	12.94 ± 2.42	<0.001
MCV (80–96 fL)	67.45 ± 8.35	80.05 ± 9.64	<0.001
MCH (26–32 pg)	22.88 ± 4.73	26.45 ± 3.70	<0.05
MCHC (30–36%)	32.69 ± 1.50	33.16 ± 1.43	>0.05
RDW (12–15%)	18.82 ± 3.45	17.99 ± 3.81	>0.05
Serum iron (40–170 mcg/dL)	17.73 ± 10.86	62.26 ± 17.60	<0.001
UIBC (110–370 mcg/dL)	429.30 ± 43.60	242.52 ± 70.17	<0.001
Ferritin	6.40 ± 4.26	114.39 ± 67.98	<0.001

IDA: iron deficiency anemia; MCV: mean corpuscular volume; MCH: mean corpuscular hemoglobin; MCHC: mean corpuscular hemoglobin concentration; RDW: red cell distribution width; UIBC: unsaturated iron-binding capacity.

**Table 3:** Comparison of thrombocyte parameters, WBC, and CRP.

	Group 1 (reactive thrombocytosis—IDA)	Group 2 (essential thrombocythemia)	*P*
WBC (4.5–11 × 10^9^/L)	9.54 ± 4.57	11.36 ± 5.61	<0.05
PLT (150–450 × 10^9^/L)	548.72 ± 72.65	973.82 ± 432.74	<0.001
MPV (7–12 fL)	7.49 ± 1.40	8.80 ± 1.50	**<0.01**
CRP (0–10 mg/L)	21.51 ± 38.36	3.96 ± 6.81	<0.01

IDA: iron deficiency anemia; WBC: white blood cell; PLT: platelet; MPV: mean platelet volume; CRP: C-reactive protein.

**Table 4:** Comparison of laboratory tests in ET group.

	JAK2 nonmutated group	JAK2 heterozygous mutant group	*P*
Hemoglobin (g/dL)	12.36 ± 2.64	13.37 ± 2.35	>0.05
PLT	1030.00 ± 466.35	961.91 ± 421.44	>0.05
MPV (7–12 fL)	8.28 ± 1.58	9.10 ± 1.36	>0.05
WBC	12.60 ± 7.47	10.61 ± 3.78	>0.05
Sex (M/F)	3/7	6/7	>0.05
Age	72.90 ± 10.46	58.08 ± 16.46	**<0.05**

PLT: platelet; MPV: mean platelet volume; WBC: white blood cell.

**Table 5:** The results of Pearson's correlation test in patients with reactive thrombocytosis (iron deficiency anemia).

Parameters	S Iron	UIBC	Ferritin	Hb	MCV	WBC	CRP
Platelet counts							
*r*	0.209	−0.230	0.135	−0.251	−0.069	−0.151	−0.089
*P*	>0.05	>0.05	>0.05	>0.05	>0.05	>0.05	>0.05
Mean platelet volume							
*r*	−0.643	0.275	−0.503	−0.389	−0.583	−0.152	−0.208
*P*	**<0.01**	>0.05	**<0.01**	**<0.05**	**<0.01**	>0.05	>0.05

S Iron: serum iron; UIBC: unsaturated iron-binding capacity; Hb: hemoglobin; MCV: mean corpuscular volume; WBC: white blood cell; CRP: C-reactive protein.

**Table 6:** The results of Pearson's correlation test in patients with essential thrombocythemia.

Parameters	S Iron	UIBC	Ferritin	Hb	MCV	WBC	CRP
Platelet counts							
*r*	0.251	0.031	0.309	−0.519	−0.459	0.702	−0.110
*P*	>0.05	>0.05	>0.05	**<0.05**	**<0.05**	**<0.01**	>0.05
Mean platelet volume							
*r*	−0.052	0.062	0.045	−0.175	−0.371	0.409	0.006
*P*	>0.05	>0.05	>0.05	>0.05	>0.05	>0.05	>0.05

S Iron: serum iron; UIBC: unsaturated iron-binding capacity; Hb: hemoglobin; MCV: mean corpuscular volume; WBC: white blood cell; CRP: C-reactive protein.
